# Complete Genome Sequence of the Gut Commensal and Laboratory Strain *Enterococcus faecium* 64/3

**DOI:** 10.1128/genomeA.01275-15

**Published:** 2015-11-19

**Authors:** Jennifer K. Bender, Stefan Fiedler, Ingo Klare, Guido Werner

**Affiliations:** National Reference Centre for Staphylococci and Enterococci, Division of Nosocomial Pathogens and Antibiotic Resistances, Department of Infectious Diseases, Robert Koch Institute, Wernigerode Branch, Germany

## Abstract

The genome sequence of the commensal and widely used laboratory strain *Enterococcus faecium* 64/3 was resolved by means of PacificBioscience and Illumina whole-genome sequencing. The genome comprises 2,575,333 bp with 2,382 coding sequences as assigned by NCBI.

## GENOME ANNOUNCEMENT

We report about the elucidation of the genome sequence of *Enterococcus faecium* 64/3, a widely used laboratory control strain and recipient for intra- and interspecies conjugation studies. *E. faecium* 64/3 (ST21) is a high-level rifampin and fusidic acid resistant derivative of a stool sample isolate from a hospital patient (MICs >128 mg/L for rifampin and fusidic acid). Other than the resistances introduced, 64/3 is susceptible to all antibiotics tested and is free of plasmids (1, 2).

The nucleotide sequence of *E. faecium* 64/3 was resolved by a commercial service provider (GATC Biotech, Konstanz, Germany) utilizing PacBio single-molecule real-time (SMRT) technology on an RS system (Pacific Biosciences, USA). Whole-genome sequencing produced a total of 88,263 reads with a mean length of 13,896 bp. Subsequent *de novo* assembly utilizing the HGAP3 protocol yielded a single polished contig with 218-fold average reference coverage. In order to ensure closed circle conformation of the bacterial chromosome, Illumina short-read sequencing was performed in-house on a MiSeq instrument and a 500-cycle v2 sequencing kit according to the manufacturer’s instructions (Illumina). Mapping and sequence analyses were carried out using the commercial software package Geneious version 7.1.4. Ring closure was further validated by PCR (not shown).

According to the NCBI annotation pipeline, 2,382 coding sequences were predicted for the chromosome of *E. faecium* 64/3. Applying the online interface ResFinder from the Center for Genomic Epidemiology (3) revealed the presence of macrolide, lincosamide, and streptogramin B resistance gene *msr(C)*. Six amino acid alterations were detected by comparing the protein sequence of Msr(C) of *E. faecium* 64/3 to a reference Msr(C) protein as provided by NCBI (AY004350).

Further, the pathogenicity factor and collagen adhesin Acm (4) was the sole virulence determinant found by utilizing the VirulenceFinder protocol from the very same Web page. A truncated N-terminus and 4 amino acid substitutions might impact Acm function in 64/3; however, this was not analyzed in more detail.

As expected for a rifampin and fusidic acid resistant strain, mutations in the respective genes were detected. For rifampin resistance, a T1465C mutation in *rpoB* results in amino acid substitution of tyrosine for histidine when compared to RpoB of reference *E. faecium* DO (CP003583.1) (5). Furthermore, two mutations were observed in *fusA* of *E. faecium* 64/3 in comparison to *E. faecium* DO. Nucleotide alterations T196G (alanine to serine) and A1366C (histidine to asparagine) most likely account for the fusidic acid resistance phenotype of *E. faecium* 64/3, as the latter mutation is located within a region which has recently been hypothesized to cause fusidic acid resistance in *E. faecalis* OG1RF (6).

No plasmid DNA was obtained by PacBio sequencing, which was confirmed by S1 nuclease–treated genomic DNA resolved in pulsed-field gel electrophoresis (not shown).

### Nucleotide sequence accession number.

The genome sequence of *E. faecium* 64/3 has been deposited in GenBank under the accession number CP012522.
